# Supplementary material for: Temperature-dependent modulation of light-induced circadian responses in Drosophila melanogaster
Source: EMBO J. 2025 Jun 30;44(16):4552–76. doi: 10.1038/s44318-025-00499-w (PMC12361518; doi:10.1038/s44318-025-00499-w)
Supplement: Supplementary file 1 — Table EV1 [file 44318_2025_499_MOESM1_ESM.pdf]

Table EV1 The list of the two-way ANOVA analysis results of Figure 2E

No	Tukey's multiple comparisons test	Predicted (LS) mean diff.	95.00% CI of diff.	Significant?	Summary	Adjusted P Value
1	Blue:ZT1 vs. Blue:ZT6	0.1169	-0.3795 to 0.6133	No	ns	0.9997
2	Blue:ZT1 vs. Blue:ZT12	-0.2006	-0.7147 to 0.3136	No	ns	0.9784
3	Blue:ZT1 vs. Blue:ZT18	-0.7636	-1.249 to -0.2779	Yes	****	<0.0001
4	Blue:ZT1 vs. Green :ZT1	0.01472	-0.1312 to 0.1606	No	ns	>0.9999
5	Blue:ZT1 vs. Yellow:ZT1	-0.008707	-0.1546 to 0.1372	No	ns	>0.9999
6	Blue:ZT6 vs. Blue:ZT12	-0.3174	-0.8189 to 0.1840	No	ns	0.6213
7	Blue:ZT6 vs. Blue:ZT18	-0.8805	-1.353 to -0.4084	Yes	****	<0.0001
8	Blue:ZT6 vs. Green :ZT6	-0.0207	-0.1593 to 0.1179	No	ns	>0.9999
9	Blue:ZT6 vs. Yellow:ZT6	-0.08104	-0.2196 to 0.05753	No	ns	0.7301
10	Blue:ZT12 vs. Blue:ZT18	-0.563	-1.054 to -0.07216	Yes	*	0.0107
11	Blue:ZT12 vs. Green :ZT12	-0.03846	-0.1870 to 0.1101	No	ns	0.9994
12	Blue:ZT12 vs. Yellow:ZT12	-0.07936	-0.2279 to 0.06922	No	ns	0.8289
13	Blue:ZT18 vs. Green :ZT18	-0.03951	-0.1717 to 0.09270	No	ns	0.9977
14	Blue:ZT18 vs. Yellow:ZT18	-0.04847	-0.1807 to 0.08374	No	ns	0.9867
15	Green :ZT1 vs. Green :ZT6	0.08146	-0.4150 to 0.5779	No	ns	>0.9999
16	Green :ZT1 vs. Green :ZT12	-0.2537	-0.7678 to 0.2604	No	ns	0.8912
17	Green :ZT1 vs. Green :ZT18	-0.8178	-1.304 to -0.3321	Yes	****	<0.0001
18	Green :ZT1 vs. Yellow:ZT1	-0.02343	-0.1693 to 0.1225	No	ns	>0.9999
19	Green :ZT6 vs. Green :ZT12	-0.3352	-0.8366 to 0.1663	No	ns	0.5374
20	Green :ZT6 vs. Green :ZT18	-0.8993	-1.371 to -0.4272	Yes	****	<0.0001
21	Green :ZT6 vs. Yellow:ZT6	-0.06034	-0.1989 to 0.07823	No	ns	0.9518
22	Green :ZT12 vs. Green :ZT18	-0.5641	-1.055 to -0.07321	Yes	*	0.0104
23	Green :ZT12 vs. Yellow:ZT12	-0.0409	-0.1895 to 0.1077	No	ns	0.9989
24	Green :ZT18 vs. Yellow:ZT18	-0.008965	-0.1412 to 0.1232	No	ns	>0.9999
25	Yellow:ZT1 vs. Yellow:ZT6	0.04455	-0.4519 to 0.5410	No	ns	>0.9999
26	Yellow:ZT1 vs. Yellow:ZT12	-0.2712	-0.7853 to 0.2429	No	ns	0.8401
27	Yellow:ZT1 vs. Yellow:ZT18	-0.8033	-1.289 to -0.3176	Yes	****	<0.0001
28	Yellow:ZT6 vs. Yellow:ZT12	-0.3158	-0.8172 to 0.1857	No	ns	0.6291
29	Yellow:ZT6 vs. Yellow:ZT18	-0.8479	-1.320 to -0.3758	Yes	****	<0.0001
30	Yellow:ZT12 vs. Yellow:ZT18	-0.5321	-1.023 to -0.04127	Yes	*	0.0213
