# Supplementary material for: Temperature-dependent modulation of light-induced circadian responses in Drosophila melanogaster
Source: EMBO J. 2025 Jun 30;44(16):4552–76. doi: 10.1038/s44318-025-00499-w (PMC12361518; doi:10.1038/s44318-025-00499-w)
Supplement: Supplementary file 2 — Table EV2 [file 44318_2025_499_MOESM2_ESM.pdf]

Table EV2 The list of the two-way ANOVA analysis results of Figure 3

Tukey's multiple comparisons test	Mean Diff.	95.00% CI of diff.	Significant?	Summary	Adjusted P Value
B *Dvpdf-LexA>GCaMP6s*					
ZT1 vs. ZT6	0.11	-0.7415 to 0.9615	No	ns	0.9849
ZT1 vs. ZT12	-0.3211	-1.173 to 0.5304	No	ns	0.7371
ZT1 vs. ZT18	-1.835	-2.626 to -1.044	Yes	****	<0.0001
ZT6 vs. ZT12	-0.4311	-1.283 to 0.4204	No	ns	0.5246
ZT6 vs. ZT18	-1.945	-2.736 to -1.154	Yes	****	<0.0001
ZT12 vs. ZT18	-1.514	-2.305 to -0.7226	Yes	****	<0.0001
B *norpA1$^{-/-}$; Dvpdf-LexA>GCaMP6s*					
ZT1 vs. ZT6	0.001743	-0.2809 to 0.2844	No	ns	>0.9999
ZT1 vs. ZT12	-0.02218	-0.3049 to 0.2605	No	ns	0.9964
ZT1 vs. ZT18	0.1242	-0.1811 to 0.4295	No	ns	0.6833
ZT6 vs. ZT12	-0.02393	-0.3066 to 0.2587	No	ns	0.9955
ZT6 vs. ZT18	0.1224	-0.1829 to 0.4277	No	ns	0.6926
ZT12 vs. ZT18	0.1464	-0.1590 to 0.4517	No	ns	0.562
C *UAS-hid; Dvpdf-LexA>GCaMP6s*					
ZT1 vs. ZT6	0.0153	-0.7459 to 0.7765	No	ns	>0.9999
ZT1 vs. ZT12	-1.061	-1.796 to -0.3255	Yes	**	0.0019
ZT1 vs. ZT18	-1.848	-2.609 to -1.087	Yes	****	<0.0001
ZT6 vs. ZT12	-1.076	-1.837 to -0.3150	Yes	**	0.0024
ZT6 vs. ZT18	-1.863	-2.649 to -1.077	Yes	****	<0.0001
ZT12 vs. ZT18	-0.787	-1.548 to -0.02584	Yes	*	0.0401
C *Rh6>hid; Dvpdf-LexA>GCaMP6s*					
ZT1 vs. ZT6	0.01784	-0.5598 to 0.5955	No	ns	0.9998
ZT1 vs. ZT12	-0.2673	-0.8449 to 0.3103	No	ns	0.593
ZT1 vs. ZT18	-0.6609	-1.239 to -0.08332	Yes	*	0.0203
ZT6 vs. ZT12	-0.2851	-0.8628 to 0.2925	No	ns	0.5413
ZT6 vs. ZT18	-0.6788	-1.256 to -0.1012	Yes	*	0.0166
ZT12 vs. ZT18	-0.3936	-0.9712 to 0.1840	No	ns	0.2676
D *Dvpdf-LexA>GCaMP6s*					
ZT1 vs. ZT6	-0.2882	-1.173 to 0.5967	No	ns	0.807
ZT1 vs. ZT12	-0.5486	-1.408 to 0.3104	No	ns	0.3171
ZT1 vs. ZT18	-1.928	-2.787 to -1.069	Yes	****	<0.0001
ZT6 vs. ZT12	-0.2604	-1.084 to 0.5628	No	ns	0.8201
ZT6 vs. ZT18	-1.64	-2.463 to -0.8168	Yes	****	<0.0001
ZT12 vs. ZT18	-1.38	-2.175 to -0.5844	Yes	***	0.0004
D *Dvpdf-LexA>GCaMP6s; cry^{02}*					
ZT1 vs. ZT6	0.4351	-0.6843 to 1.555	No	ns	0.7074
ZT1 vs. ZT12	-0.3487	-1.545 to 0.8480	No	ns	0.8507

ZT1 vs. ZT18	-1.992	-3.145 to -0.8387	Yes	***	0.0004
ZT6 vs. ZT12	-0.7839	-1.903 to 0.3356	No	ns	0.2406
ZT6 vs. ZT18	-2.427	-3.500 to -1.354	Yes	****	<0.0001
ZT12 vs. ZT18	-1.643	-2.796 to -0.4899	Yes	**	0.0034
