# Supplementary material for: Temperature-dependent modulation of light-induced circadian responses in Drosophila melanogaster
Source: EMBO J. 2025 Jun 30;44(16):4552–76. doi: 10.1038/s44318-025-00499-w (PMC12361518; doi:10.1038/s44318-025-00499-w)
Supplement: Supplementary file 3 — Table EV3 [file 44318_2025_499_MOESM3_ESM.pdf]

Table EV3 The list of the two-way ANOVA analysis results of Figure 4

Tukey's multiple comparisons test	Mean Diff.	95.00% CI of diff.	Significant?	Summary	Adjusted P Value
**B *Dvpdf-LexA>GCaMP6s***					
ZT1 vs. ZT6	0.11	-0.7415 to 0.9615	No	ns	0.9849
ZT1 vs. ZT12	-0.3211	-1.173 to 0.5304	No	ns	0.7371
ZT1 vs. ZT18	-1.835	-2.626 to -1.044	Yes	****	<0.0001
ZT6 vs. ZT12	-0.4311	-1.283 to 0.4204	No	ns	0.5246
ZT6 vs. ZT18	-1.945	-2.736 to -1.154	Yes	****	<0.0001
ZT12 vs. ZT18	-1.514	-2.305 to -0.7226	Yes	****	<0.0001
**B *han*^{5304}*; Dvpdf-LexA>GCaMP6s***					
ZT1 vs. ZT6	-0.06561	-1.231 to 1.100	No	ns	0.9987
ZT1 vs. ZT12	-1.166	-2.331 to - 0.0009594	Yes	*	0.0498
ZT1 vs. ZT18	-1.772	-2.878 to -0.6670	Yes	***	0.0008
ZT6 vs. ZT12	-1.101	-2.266 to 0.06465	No	ns	0.0695
ZT6 vs. ZT18	-1.707	-2.812 to -0.6014	Yes	**	0.0012
ZT12 vs. ZT18	-0.6062	-1.712 to 0.4992	No	ns	0.4551
**D *Dvpdf-LexA>GCaMP6s***					
ZT1 vs. ZT6	0.11	-0.7415 to 0.9615	No	ns	0.9849
ZT1 vs. ZT12	-0.3211	-1.173 to 0.5304	No	ns	0.7371
ZT1 vs. ZT18	-1.835	-2.626 to -1.044	Yes	****	<0.0001
ZT6 vs. ZT12	-0.4311	-1.283 to 0.4204	No	ns	0.5246
ZT6 vs. ZT18	-1.945	-2.736 to -1.154	Yes	****	<0.0001
ZT12 vs. ZT18	-1.514	-2.305 to -0.7226	Yes	****	<0.0001
**D *per*^{0}*; Dvpdf-LexA>GCaMP6s***					
ZT1 vs. ZT6	0.1136	-0.9438 to 1.171	No	ns	0.9907
ZT1 vs. ZT12	-0.0277	-1.158 to 1.103	No	ns	0.9999
ZT1 vs. ZT18	0.03856	-1.019 to 1.096	No	ns	0.9996
ZT6 vs. ZT12	-0.1413	-1.199 to 0.9161	No	ns	0.9825
ZT6 vs. ZT18	-0.07501	-1.054 to 0.9039	No	ns	0.9966
ZT12 vs. ZT18	0.06626	-0.9911 to 1.124	No	ns	0.9981
**F *Dvpdf-LexA>GCaMP6s***					
ZT1 vs. ZT6	-0.05587	-0.9860 to 0.8742	No	ns	0.9983
ZT1 vs. ZT12	-0.3795	-1.491 to 0.7322	No	ns	0.7796
ZT1 vs. ZT18	-1.43	-2.361 to -0.5004	Yes	**	0.0016
ZT6 vs. ZT12	-0.3236	-1.378 to 0.7310	No	ns	0.829
ZT6 vs. ZT18	-1.375	-2.236 to -0.5135	Yes	**	0.0011
ZT12 vs. ZT18	-1.051	-2.106 to 0.003663	No	ns	0.051
**F *per*^{0}*; Dvpdf-LexA>GCaMP6s***					
ZT1 vs. ZT6	-0.4752	-0.9566 to	No	ns	0.0538

		0.006055			
ZT1 vs. ZT12	-0.07506	-0.5896 to 0.4395	No	ns	0.9774
ZT1 vs. ZT18	-0.1995	-0.6808 to 0.2818	No	ns	0.6669
ZT6 vs. ZT12	0.4002	-0.08112 to 0.8815	No	ns	0.1277
ZT6 vs. ZT18	0.2757	-0.1699 to 0.7213	No	ns	0.3422
ZT12 vs. ZT18	-0.1245	-0.6058 to 0.3568	No	ns	0.8908
