# Supplementary material for: Temperature-dependent modulation of light-induced circadian responses in Drosophila melanogaster
Source: EMBO J. 2025 Jun 30;44(16):4552–76. doi: 10.1038/s44318-025-00499-w (PMC12361518; doi:10.1038/s44318-025-00499-w)
Supplement: Supplementary file 4 — Table EV4 [file 44318_2025_499_MOESM4_ESM.pdf]

Table EV4 The list of the two-way ANOVA analysis results of Figure 6

Tukey's multiple comparisons test	Mean Diff.	95.00% CI of diff.	Significant?	Summary	Adjusted P Value
**E *hid;Dvpdf-LexA>GCaMP6s***					
ZT1 vs. ZT6	0.0135	-0.7492 to 0.7762	No	ns	>0.9999
ZT1 vs. ZT12	-1.035	-1.771 to -0.2978	Yes	**	0.0026
ZT1 vs. ZT18	-1.817	-2.580 to -1.054	Yes	****	<0.0001
ZT6 vs. ZT12	-1.048	-1.811 to -0.2854	Yes	**	0.0033
ZT6 vs. ZT18	-1.831	-2.618 to -1.043	Yes	****	<0.0001
ZT12 vs. ZT18	-0.7825	-1.545 to -0.01981	Yes	*	0.0423
**E *Dvpdf-LexA>GCaMP6s; DN1a>hid***					
ZT1 vs. ZT6	0.06337	-0.6242 to 0.7510	No	ns	0.9947
ZT1 vs. ZT12	-0.303	-1.024 to 0.4181	No	ns	0.6802
ZT1 vs. ZT18	-0.8888	-1.517 to -0.2611	Yes	**	0.0025
ZT6 vs. ZT12	-0.3664	-1.088 to 0.3548	No	ns	0.535
ZT6 vs. ZT18	-0.9522	-1.580 to -0.3245	Yes	**	0.0011
ZT12 vs. ZT18	-0.5858	-1.250 to 0.07849	No	ns	0.1017
**F *vGlutRNAi; Dvpdf-LexA>GCaMP6s***					
ZT1 vs. ZT6	-0.1356	-0.9814 to 0.7101	No	ns	0.9741
ZT1 vs. ZT12	-0.9405	-1.878 to -0.003563	Yes	*	0.0488
ZT1 vs. ZT18	-1.085	-1.897 to -0.2731	Yes	**	0.0044
ZT6 vs. ZT12	-0.8049	-1.742 to 0.1321	No	ns	0.1165
ZT6 vs. ZT18	-0.9492	-1.761 to -0.1375	Yes	*	0.0157
ZT12 vs. ZT18	-0.1443	-1.051 to 0.7621	No	ns	0.9747
**F *Dicer;Dvpdf-LexA>GCaMP6s; DN1a>vGlutRNAi***					
ZT1 vs. ZT6	-0.1891	-0.9069 to 0.5287	No	ns	0.8976
ZT1 vs. ZT12	-0.2322	-0.9500 to 0.4856	No	ns	0.827
ZT1 vs. ZT18	-1.108	-1.883 to -0.3328	Yes	**	0.0021
ZT6 vs. ZT12	-0.04309	-0.7609 to 0.6747	No	ns	0.9986
ZT6 vs. ZT18	-0.9189	-1.694 to -0.1437	Yes	*	0.014
ZT12 vs. ZT18	-0.8758	-1.651 to -0.1006	Yes	*	0.0209
