# Supplementary material for: Temperature-dependent modulation of light-induced circadian responses in Drosophila melanogaster
Source: EMBO J. 2025 Jun 30;44(16):4552–76. doi: 10.1038/s44318-025-00499-w (PMC12361518; doi:10.1038/s44318-025-00499-w)
Supplement: Supplementary file 5 — Table EV5 [file 44318_2025_499_MOESM5_ESM.pdf]

Table EV5 The list of the three-way ANOVA analysis results of Figure 7B

No	Tukey's multiple comparisons test	Mean Diff.	95.00% CI of diff.	Significant?	Summary	Adjusted P Value
1	D1:*cry*^{02} 24℃ vs. D1:*cry*^{02} 29℃	2.214	1.134 to 3.294	Yes	****	<0.0001
2	D1:*cry*^{02} 24℃ vs. D1:*w*^{1118} 24℃	0.75	-0.1951 to 1.695	No	ns	0.3397
3	D1:*cry*^{02} 29℃ vs. D1:*w*^{1118} 29℃	-0.4643	-1.406 to 0.4770	No	ns	0.9696
4	D1:*w*^{1118} 24℃ vs. D1:*w*^{1118} 29℃	1	0.2174 to 1.783	Yes	**	0.0011
5	D2:*cry*^{02} 24℃ vs. D2:*cry*^{02} 29℃	2.413	1.333 to 3.493	Yes	****	<0.0001
6	D2:*cry*^{02} 24℃ vs. D2:*w*^{1118} 24℃	-2.236	-3.181 to -1.291	Yes	****	<0.0001
7	D2:*cry*^{02} 29℃ vs. D2:*w*^{1118} 29℃	-4.77	-5.711 to -3.828	Yes	****	<0.0001
8	D2:*w*^{1118} 24℃ vs. D2:*w*^{1118} 29℃	-0.1208	-0.9035 to 0.6618	No	ns	>0.9999
9	D3:*cry*^{02} 24℃ vs. D3:*cry*^{02} 29℃	1.754	0.6738 to 2.834	Yes	****	<0.0001
10	D3:*cry*^{02} 24℃ vs. D3:*w*^{1118} 24℃	-0.8472	-1.792 to 0.09789	No	ns	0.1463
11	D3:*cry*^{02} 29℃ vs. D3:*w*^{1118} 29℃	-2.78	-3.722 to -1.839	Yes	****	<0.0001
12	D3:*w*^{1118} 24℃ vs. D3:*w*^{1118} 29℃	-0.1792	-0.9618 to 0.6035	No	ns	>0.9999
13	D4:*cry*^{02} 24℃ vs. D4:*cry*^{02} 29℃	1.448	0.3683 to 2.529	Yes	***	0.0004
14	D4:*cry*^{02} 24℃ vs. D4:*w*^{1118} 24℃	-0.006944	-0.9521 to 0.9382	No	ns	>0.9999
15	D4:*cry*^{02} 29℃ vs. D4:*w*^{1118} 29℃	-1.605	-2.547 to -0.6641	Yes	****	<0.0001
16	D4:*w*^{1118} 24℃ vs. D4:*w*^{1118} 29℃	0.075	-0.7076 to 0.8576	No	ns	>0.9999
17	D5:*cry*^{02} 24℃ vs. D5:*cry*^{02} 29℃	0.9286	-0.1516 to 2.009	No	ns	0.2031
18	D5:*cry*^{02} 24℃ vs. D5:*w*^{1118} 24℃	0.3125	-0.6326 to 1.258	No	ns	0.9998
19	D5:*cry*^{02} 29℃ vs. D5:*w*^{1118} 29℃	-0.6661	-1.607 to 0.2752	No	ns	0.5669
20	D5:*w*^{1118} 24℃ vs. D5:*w*^{1118} 29℃	-0.05	-0.8326 to 0.7326	No	ns	>0.9999
21	D1:*cry*^{02} 24℃ vs. D1:*cs* 24℃	0.8824	-0.2887 to 2.053	No	ns	0.4403
22	D1:*cry*^{02} 29℃ vs. D1:*cs* 29℃	-0.9048	-2.291 to 0.4814	No	ns	0.7136
23	D1:*cs* 24℃ vs. D1:*cs* 29℃	0.4272	-0.6879 to 1.542	No	ns	0.9984
24	D2:*cry*^{02} 24℃ vs. D2:*cs* 24℃	-2.092	-3.263 to -0.9204	Yes	****	<0.0001
25	D2:*cry*^{02} 29℃ vs. D2:*cs* 29℃	-5.119	-6.505 to -3.733	Yes	****	<0.0001
26	D2:*cs* 24℃ vs. D2:*cs* 29℃	-0.6148	-1.730 to 0.5002	No	ns	0.9139
27	D3:*cry*^{02} 24℃ vs. D3:*cs* 24℃	-0.8154	-1.986 to 0.3557	No	ns	0.5973
28	D3:*cry*^{02} 29℃ vs. D3:*cs* 29℃	-3.107	-4.493 to -1.721	Yes	****	<0.0001
29	D3:*cs* 24℃ vs. D3:*cs* 29℃	-0.4902	-1.605 to 0.6248	No	ns	0.9912
30	D4:*cry*^{02} 24℃ vs. D4:*cs* 24℃	-0.03268	-1.204 to 1.138	No	ns	>0.9999
31	D4:*cry*^{02} 29℃ vs. D4:*cs* 29℃	-2.06	-3.446 to -0.6734	Yes	****	<0.0001
32	D4:*cs* 24℃ vs. D4:*cs* 29℃	-0.5784	-1.693 to 0.5366	No	ns	0.9499
33	D5:*cry*^{02} 24℃ vs. D5:*cs* 24℃	0.1618	-1.009 to 1.333	No	ns	>0.9999
34	D5:*cry*^{02} 29℃ vs. D5:*cs* 29℃	-1.488	-2.874 to -0.1019	Yes	*	0.0207
35	D5:*cs* 24℃ vs. D5:*cs* 29℃	-0.7213	-1.836 to 0.3937	No	ns	0.7283
