# Supplementary material for: Temperature-dependent modulation of light-induced circadian responses in Drosophila melanogaster
Source: EMBO J. 2025 Jun 30;44(16):4552–76. doi: 10.1038/s44318-025-00499-w (PMC12361518; doi:10.1038/s44318-025-00499-w)
Supplement: Supplementary file 6 — Table EV6 [file 44318_2025_499_MOESM6_ESM.pdf]

Table EV6 The list of the three-way ANOVA analysis results of Figure 7C

No	Tukey's multiple comparisons test	Mean Diff.	95.00% CI of diff.	Significant?	Summary	Adjusted P Value
1	D1:*cry*^{02} 24℃ vs. D1:*cry*^{02} 19℃	-0.5833	-1.489 to 0.3221	No	ns	0.7369
2	D1:*cry*^{02} 24℃ vs. D1:*w*^{1118} 24℃	0.75	-0.1970 to 1.697	No	ns	0.344
3	D1:*cry*^{02} 19℃ vs. D1:*w*^{1118} 19℃	0.6302	-0.1416 to 1.402	No	ns	0.2879
4	D1:*w*^{1118} 24℃ vs. D1:*w*^{1118} 19℃	-0.7031	-1.523 to 0.1170	No	ns	0.2075
5	D2:*cry*^{02} 24℃ vs. D2:*cry*^{02} 19℃	-0.6111	-1.517 to 0.2944	No	ns	0.6576
6	D2:*cry*^{02} 24℃ vs. D2:*w*^{1118} 24℃	-2.236	-3.183 to -1.289	Yes	****	<0.0001
7	D2:*cry*^{02} 19℃ vs. D2:*w*^{1118} 19℃	-1.677	-2.449 to -0.9053	Yes	****	<0.0001
8	D2:*w*^{1118} 24℃ vs. D2:*w*^{1118} 19℃	-0.05208	-0.8722 to 0.7680	No	ns	>0.9999
9	D3:*cry*^{02} 24℃ vs. D3:*cry*^{02} 19℃	0.3278	-0.5777 to 1.233	No	ns	0.9993
10	D3:*cry*^{02} 24℃ vs. D3:*w*^{1118} 24℃	-0.8472	-1.794 to 0.09975	No	ns	0.1489
11	D3:*cry*^{02} 19℃ vs. D3:*w*^{1118} 19℃	-1.342	-2.113 to -0.5699	Yes	****	<0.0001
12	D3:*w*^{1118} 24℃ vs. D3:*w*^{1118} 19℃	-0.1667	-0.9868 to 0.6534	No	ns	>0.9999
13	D4:*cry*^{02} 24℃ vs. D4:*cry*^{02} 19℃	0.4722	-0.4333 to 1.378	No	ns	0.9482
14	D4:*cry*^{02} 24℃ vs. D4:*w*^{1118} 24℃	-0.006944	-0.9539 to 0.9400	No	ns	>0.9999
15	D4:*cry*^{02} 19℃ vs. D4:*w*^{1118} 19℃	-0.7135	-1.485 to 0.05827	No	ns	0.1118
16	D4:*w*^{1118} 24℃ vs. D4:*w*^{1118} 19℃	-0.2344	-1.054 to 0.5857	No	ns	>0.9999
17	D5:*cry*^{02} 24℃ vs. D5:*cry*^{02} 19℃	0.6	-0.3055 to 1.505	No	ns	0.6901
18	D5:*cry*^{02} 24℃ vs. D5:*w*^{1118} 24℃	0.3125	-0.6345 to 1.259	No	ns	0.9998
19	D5:*cry*^{02} 19℃ vs. D5:*w*^{1118} 19℃	-0.5844	-1.356 to 0.1874	No	ns	0.4322
20	D5:*w*^{1118} 24℃ vs. D5:*w*^{1118} 19℃	-0.2969	-1.117 to 0.5232	No	ns	0.9993
21	D1:*cry*^{02} 24℃ vs. D1:*cs* 24℃	0.8824	-0.2739 to 2.039	No	ns	0.4172
22	D1:*cry*^{02} 19℃ vs. D1:*cs* 19℃	0.5349	-0.4809 to 1.551	No	ns	0.9437
23	D1:*cs* 24℃ vs. D1:*cs* 19℃	-0.9307	-1.916 to 0.05432	No	ns	0.091
24	D2:*cry*^{02} 24℃ vs. D2:*cs* 24℃	-2.092	-3.248 to -0.9353	Yes	****	<0.0001
25	D2:*cry*^{02} 19℃ vs. D2:*cs* 19℃	-1.946	-2.962 to -0.9303	Yes	****	<0.0001
26	D2:*cs* 24℃ vs. D2:*cs* 19℃	-0.4658	-1.451 to 0.5192	No	ns	0.9807
27	D3:*cry*^{02} 24℃ vs. D3:*cs* 24℃	-0.8154	-1.972 to 0.3409	No	ns	0.5754
28	D3:*cry*^{02} 19℃ vs. D3:*cs* 19℃	-1.402	-2.418 to -0.3863	Yes	***	0.0002
29	D3:*cs* 24℃ vs. D3:*cs* 19℃	-0.259	-1.244 to 0.7260	No	ns	>0.9999
30	D4:*cry*^{02} 24℃ vs. D4:*cs* 24℃	-0.03268	-1.189 to 1.124	No	ns	>0.9999
31	D4:*cry*^{02} 19℃ vs. D4:*cs* 19℃	-0.8683	-1.884 to 0.1476	No	ns	0.2125
32	D4:*cs* 24℃ vs. D4:*cs* 19℃	-0.3634	-1.348 to 0.6217	No	ns	0.9991
33	D5:*cry*^{02} 24℃ vs. D5:*cs* 24℃	0.1618	-0.9945 to 1.318	No	ns	>0.9999
34	D5:*cry*^{02} 19℃ vs. D5:*cs* 19℃	-0.7694	-1.785 to 0.2465	No	ns	0.4325
35	D5:*cs* 24℃ vs. D5:*cs* 19℃	-0.3311	-1.316 to 0.6539	No	ns	0.9997
