# Supplementary material for: Temperature-dependent modulation of light-induced circadian responses in Drosophila melanogaster
Source: EMBO J. 2025 Jun 30;44(16):4552–76. doi: 10.1038/s44318-025-00499-w (PMC12361518; doi:10.1038/s44318-025-00499-w)
Supplement: Supplementary file 7 — Table EV7 [file 44318_2025_499_MOESM7_ESM.pdf]

Table EV7 The list of the three-way ANOVA analysis results of Figure 7D

No	Tukey's multiple comparisons test	Mean Diff.	95.00% CI of diff.	Significant?	Summary	Adjusted P Value
1	D1:cry^{02} 24℃ vs. D1:cry^{02} 29℃	0.6103	-0.9924 to 2.213	No	ns	0.9985
2	D1:cry^{02} 24℃ vs. D1:w^{1118} 24℃	4.557	3.207 to 5.908	Yes	****	<0.0001
3	D1:cry^{02} 29℃ vs. D1:w^{1118} 29℃	5.45	3.655 to 7.245	Yes	****	<0.0001
4	D1:w^{1118} 24℃ vs. D1:w^{1118} 29℃	1.503	-0.07119 to 3.077	No	ns	0.082
5	D2:cry^{02} 24℃ vs. D2:cry^{02} 29℃	-2.369	-3.971 to -0.7658	Yes	****	<0.0001
6	D2:cry^{02} 24℃ vs. D2:w^{1118} 24℃	1.003	-0.3480 to 2.353	No	ns	0.472
7	D2:cry^{02} 29℃ vs. D2:w^{1118} 29℃	3.624	1.829 to 5.419	Yes	****	<0.0001
8	D2:w^{1118} 24℃ vs. D2:w^{1118} 29℃	0.253	-1.321 to 1.827	No	ns	>0.9999
9	D3:cry^{02} 24℃ vs. D3:cry^{02} 29℃	-2.418	-4.044 to -0.7921	Yes	****	<0.0001
10	D3:cry^{02} 24℃ vs. D3:w^{1118} 24℃	0.6556	-0.6950 to 2.006	No	ns	0.9744
11	D3:cry^{02} 29℃ vs. D3:w^{1118} 29℃	3.017	1.201 to 4.833	Yes	****	<0.0001
12	D3:w^{1118} 24℃ vs. D3:w^{1118} 29℃	-0.05655	-1.631 to 1.518	No	ns	>0.9999
13	D4:cry^{02} 24℃ vs. D4:cry^{02} 29℃	-2.322	-4.652 to 0.008514	No	ns	0.0521
14	D4:cry^{02} 24℃ vs. D4:w^{1118} 24℃	0.4	-2.351 to 3.151	No	ns	>0.9999
15	D4:cry^{02} 29℃ vs. D4:w^{1118} 29℃	2.584	0.7891 to 4.379	Yes	****	<0.0001
16	D4:w^{1118} 24℃ vs. D4:w^{1118} 29℃	-0.1375	-2.453 to 2.178	No	ns	>0.9999
17	D5:cry^{02} 24℃ vs. D5:cry^{02} 29℃	-1.707	-4.037 to 0.6237	No	ns	0.4994
18	D5:cry^{02} 24℃ vs. D5:w^{1118} 24℃	0.55	-2.201 to 3.301	No	ns	>0.9999
19	D5:cry^{02} 29℃ vs. D5:w^{1118} 29℃	0.8732	-0.9219 to 2.668	No	ns	0.9738
20	D5:w^{1118} 24℃ vs. D5:w^{1118} 29℃	-1.383	-3.699 to 0.9322	No	ns	0.8405
21	D1:cry^{02} 24℃ vs. D1:*cs* 24℃	4.31	2.931 to 5.689	Yes	****	<0.0001
22	D1:cry^{02} 29℃ vs. D1:*cs* 29℃	3.571	1.474 to 5.668	Yes	****	<0.0001
23	D1:*cs* 24℃ vs. D1:*cs* 29℃	-0.1287	-2.024 to 1.766	No	ns	>0.9999
24	D2:cry^{02} 24℃ vs. D2:*cs* 24℃	1.028	-0.3510 to 2.408	No	ns	0.4626
25	D2:cry^{02} 29℃ vs. D2:*cs* 29℃	3.445	1.348 to 5.542	Yes	****	<0.0001
26	D2:*cs* 24℃ vs. D2:*cs* 29℃	0.04806	-1.847 to 1.943	No	ns	>0.9999
27	D3:cry^{02} 24℃ vs. D3:*cs* 24℃	0.711	-0.6683 to 2.090	No	ns	0.9533
28	D3:cry^{02} 29℃ vs. D3:*cs* 29℃	2.988	0.8719 to 5.104	Yes	***	0.0001
29	D3:*cs* 24℃ vs. D3:*cs* 29℃	-0.1411	-2.036 to 1.754	No	ns	>0.9999
30	D4:cry^{02} 24℃ vs. D4:*cs* 24℃	0.3364	-2.425 to 3.097	No	ns	>0.9999
31	D4:cry^{02} 29℃ vs. D4:*cs* 29℃	2.522	0.4245 to 4.619	Yes	**	0.0036
32	D4:*cs* 24℃ vs. D4:*cs* 29℃	-0.1364	-2.645 to 2.372	No	ns	>0.9999
33	D5:cry^{02} 24℃ vs. D5:*cs* 24℃	0.6136	-2.147 to 3.375	No	ns	>0.9999
34	D5:cry^{02} 29℃ vs. D5:*cs* 29℃	2.023	-0.07402 to 4.120	No	ns	0.0738
35	D5:*cs* 24℃ vs. D5:*cs* 29℃	-0.297	-2.805 to 2.211	No	ns	>0.9999
