# Supplementary material for: Temperature-dependent modulation of light-induced circadian responses in Drosophila melanogaster
Source: EMBO J. 2025 Jun 30;44(16):4552–76. doi: 10.1038/s44318-025-00499-w (PMC12361518; doi:10.1038/s44318-025-00499-w)
Supplement: Supplementary file 8 — Table EV8 [file 44318_2025_499_MOESM8_ESM.pdf]

Table EV8 The list of the three-way ANOVA analysis results of Figure 7E

No	Tukey's multiple comparisons test	Mean Diff.	95.00% CI of diff.	Significant?	Summary	Adjusted P Value
1	D1:*cry*^{02} 24℃ vs. D1:*cry*^{02} 19℃	4.68	3.780 to 5.581	Yes	****	<0.0001
2	D1:*cry*^{02} 24℃ vs. D1:*w*^{1118} 24℃	4.557	3.719 to 5.396	Yes	****	<0.0001
3	D1:*cry*^{02} 19℃ vs. D1:*w*^{1118} 19℃	-0.1563	-1.111 to 0.7984	No	ns	>0.9999
4	D1:*w*^{1118} 24℃ vs. D1:*w*^{1118} 19℃	-0.03348	-0.9295 to 0.8625	No	ns	>0.9999
5	D2:*cry*^{02} 24℃ vs. D2:*cry*^{02} 19℃	0.1254	-0.7753 to 1.026	No	ns	>0.9999
6	D2:*cry*^{02} 24℃ vs. D2:*w*^{1118} 24℃	1.003	0.1643 to 1.841	Yes	**	0.0039
7	D2:*cry*^{02} 19℃ vs. D2:*w*^{1118} 19℃	0.8906	-0.06398 to 1.845	No	ns	0.1029
8	D2:*w*^{1118} 24℃ vs. D2:*w*^{1118} 19℃	0.01339	-0.8826 to 0.9094	No	ns	>0.9999
9	D3:*cry*^{02} 24℃ vs. D3:*cry*^{02} 19℃	-0.2447	-1.145 to 0.6560	No	ns	>0.9999
10	D3:*cry*^{02} 24℃ vs. D3:*w*^{1118} 24℃	0.6556	-0.1827 to 1.494	No	ns	0.3693
11	D3:*cry*^{02} 19℃ vs. D3:*w*^{1118} 19℃	0.8281	-0.1265 to 1.783	No	ns	0.1906
12	D3:*w*^{1118} 24℃ vs. D3:*w*^{1118} 19℃	-0.07217	-0.9682 to 0.8238	No	ns	>0.9999
13	D4:*cry*^{02} 24℃ vs. D4:*cry*^{02} 19℃	-0.3313	-1.715 to 1.052	No	ns	>0.9999
14	D4:*cry*^{02} 24℃ vs. D4:*w*^{1118} 24℃	0.4	-1.308 to 2.108	No	ns	>0.9999
15	D4:*cry*^{02} 19℃ vs. D4:*w*^{1118} 19℃	0.5156	-0.4390 to 1.470	No	ns	0.9296
16	D4:*w*^{1118} 24℃ vs. D4:*w*^{1118} 19℃	-0.2156	-1.599 to 1.168	No	ns	>0.9999
17	D5:*cry*^{02} 24℃ vs. D5:*cry*^{02} 19℃	-0.5313	-1.915 to 0.8521	No	ns	0.9984
18	D5:*cry*^{02} 24℃ vs. D5:*w*^{1118} 24℃	0.55	-1.158 to 2.258	No	ns	0.9999
19	D5:*cry*^{02} 19℃ vs. D5:*w*^{1118} 19℃	0.7656	-0.1890 to 1.720	No	ns	0.3213
20	D5:*w*^{1118} 24℃ vs. D5:*w*^{1118} 19℃	-0.3156	-1.699 to 1.068	No	ns	>0.9999
21	D1:*cry*^{02} 24℃ vs. D1:*cs* 24℃	4.31	3.476 to 5.144	Yes	****	<0.0001
22	D1:*cry*^{02} 19℃ vs. D1:*cs* 19℃	0.4375	-0.5174 to 1.392	No	ns	0.9864
23	D1:*cs* 24℃ vs. D1:*cs* 19℃	0.8078	-0.08398 to 1.700	No	ns	0.1343
24	D2:*cry*^{02} 24℃ vs. D2:*cs* 24℃	1.028	0.1946 to 1.862	Yes	**	0.0022
25	D2:*cry*^{02} 19℃ vs. D2:*cs* 19℃	0.875	-0.07991 to 1.830	No	ns	0.1214
26	D2:*cs* 24℃ vs. D2:*cs* 19℃	-0.02798	-0.9197 to 0.8638	No	ns	>0.9999
27	D3:*cry*^{02} 24℃ vs. D3:*cs* 24℃	0.711	-0.1227 to 1.545	No	ns	0.2163
28	D3:*cry*^{02} 19℃ vs. D3:*cs* 19℃	0.6563	-0.2987 to 1.611	No	ns	0.6257
29	D3:*cs* 24℃ vs. D3:*cs* 19℃	-0.2994	-1.191 to 0.5923	No	ns	0.9997
30	D4:*cry*^{02} 24℃ vs. D4:*cs* 24℃	0.3364	-1.333 to 2.005	No	ns	>0.9999
31	D4:*cry*^{02} 19℃ vs. D4:*cs* 19℃	0.4375	-0.5174 to 1.392	No	ns	0.9864
32	D4:*cs* 24℃ vs. D4:*cs* 19℃	-0.2301	-1.565 to 1.105	No	ns	>0.9999
33	D5:*cry*^{02} 24℃ vs. D5:*cs* 24℃	0.6136	-1.055 to 2.283	No	ns	0.9991
34	D5:*cry*^{02} 19℃ vs. D5:*cs* 19℃	0.625	-0.3299 to 1.580	No	ns	0.7127
35	D5:*cs* 24℃ vs. D5:*cs* 19℃	-0.5199	-1.855 to 0.8151	No	ns	0.9981
