# Supplementary material for: Temperature-dependent modulation of light-induced circadian responses in Drosophila melanogaster
Source: EMBO J. 2025 Jun 30;44(16):4552–76. doi: 10.1038/s44318-025-00499-w (PMC12361518; doi:10.1038/s44318-025-00499-w)
Supplement: Supplementary file 10 — Movie EV1 [file 44318_2025_499_MOESM10_ESM.zip › Movie EV1.docx]

Light response of s-LNv fibers at ZT6, related to Figure 2
