# Supplementary material for: Temperature-dependent modulation of light-induced circadian responses in Drosophila melanogaster
Source: EMBO J. 2025 Jun 30;44(16):4552–76. doi: 10.1038/s44318-025-00499-w (PMC12361518; doi:10.1038/s44318-025-00499-w)
Supplement: Supplementary file 11 — Movie EV2 [file 44318_2025_499_MOESM11_ESM.zip › Movie EV2.docx]

Light response of s-LNv fibers at ZT18, related to Figure 2
