# Supplementary material for: Temperature-dependent modulation of light-induced circadian responses in Drosophila melanogaster
Source: EMBO J. 2025 Jun 30;44(16):4552–76. doi: 10.1038/s44318-025-00499-w (PMC12361518; doi:10.1038/s44318-025-00499-w)
Supplement: Supplementary file 20 — Expanded View Figures [file 44318_2025_499_MOESM20_ESM.pdf]

Expanded View Figures

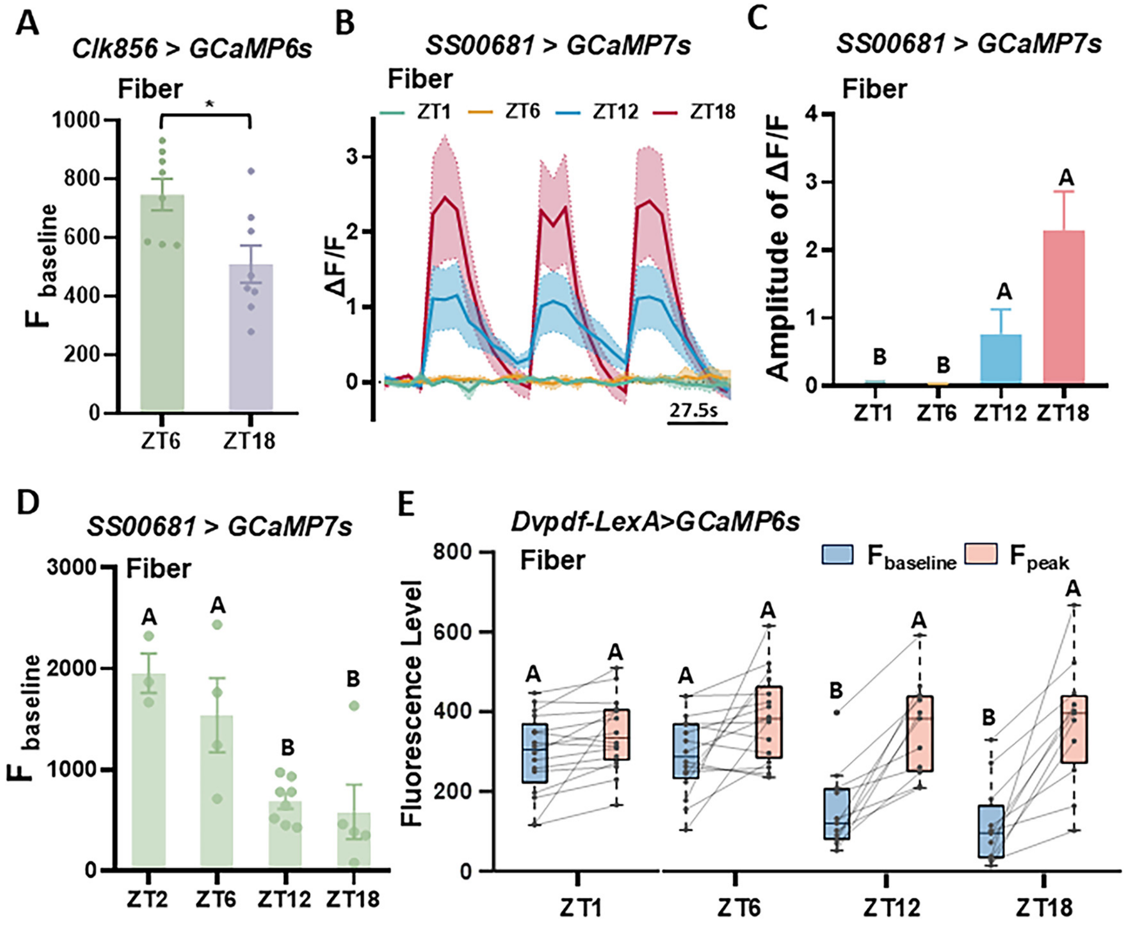

Figure EV1. Basal calcium activity level.

(**A**) Basal calcium activity level of circadian neuron dorsal fibers expressing GCaMP labeled by *CLK856-GAL4*. *N* = 4 for both ZT6 and ZT18. *P* = 0.0127. (**B**) Traces of ΔF/F_0 of s-LNvs labeled by a split-GAL4 driver at different time points in response to light pulse. (**C**) Statistical quantification of the response amplitudes of s-LNvs at different time points. *N* = 3 for ZT1, ZT6, and ZT18, and *N* = 4 for ZT12. (**D**) Basal calcium activity level of the s-LNvs dorsal fibers labeled by specific split-gal4. *N* = 3 for ZT1, ZT6, and ZT18, and *N* = 4 for ZT12. (**E**) The basal calcium fluorescence $F_{baseline}$ (blue) and maximum calcium fluorescence F_{peak} (pink) of s-LNv dorsal fibers at different time points in wild-type control flies. Data information: Data are presented as means ± SEM. *$P < 0.05$. Analyses of two samples employed a two-tailed unpaired Student's *t* test for A. One-way ANOVA with the Tukey correction to the *P* value was used to assess the significance for multiple samples in (**C**, **D**, **E**). The letters A and B above or below the curves denote significantly different means within each of the two groups, $P < 0.05$.

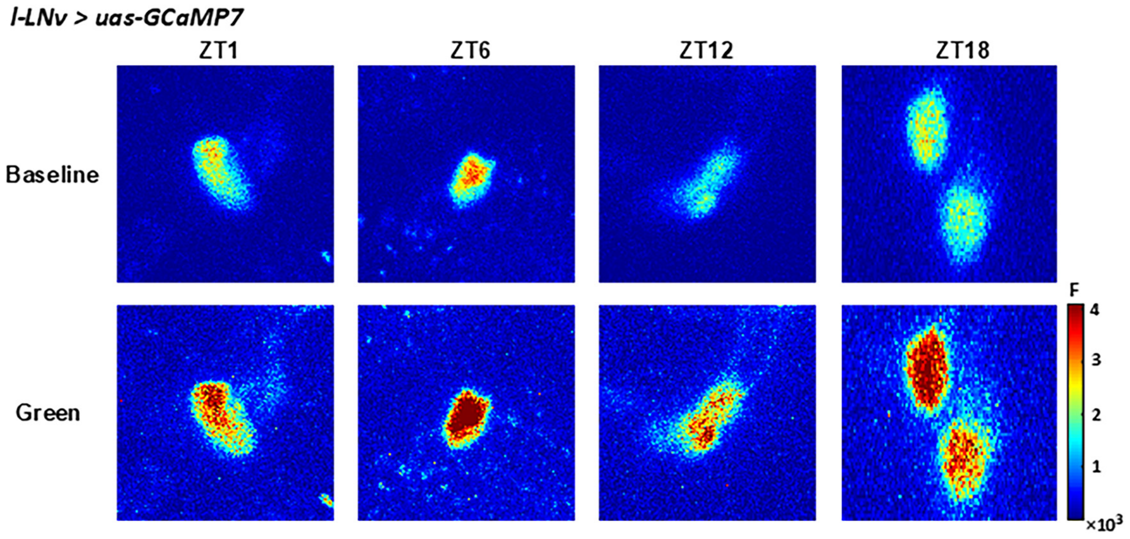

Figure EV2. Light response of l-LNv soma.

From left to right panels: Pseudocolor maps of the basal calcium activity (top row) and calcium activity changes in response to green LED stimulation (bottom row) at ZT1, ZT6, ZT12, and ZT18. Warmer colors indicate higher calcium levels. Color scales are shared across panels.

© The Author(s)

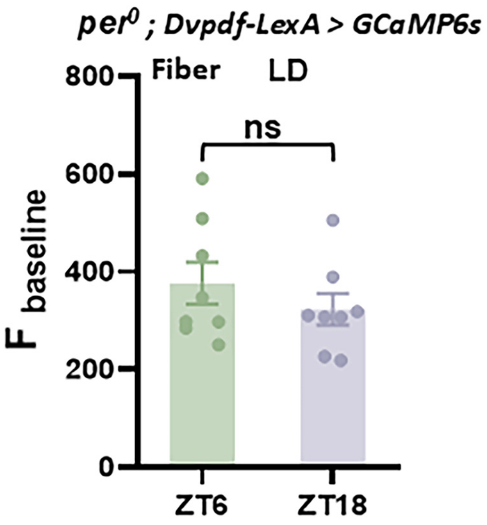

Figure EV3. Basal calcium activity level of the s-LNvs dorsal fibers in *per*^{0} mutant flies under LD.

N = 4 for both ZT6 and ZT18. Data information: Data are presented as means ± SEM. Analysis of two samples employed a two-tailed unpaired Student's *t* test.

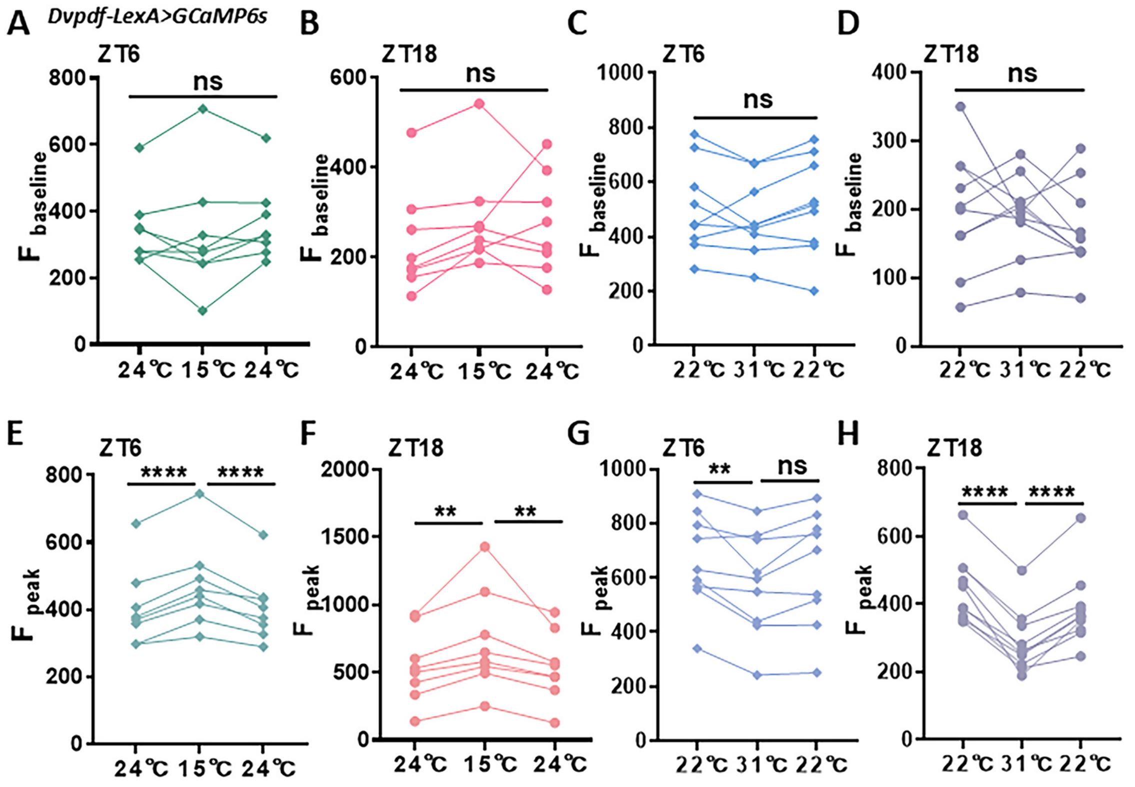

Figure EV4. Temperature-induced changes of light responses in s-LNvs.

(**A**, **B**) The basal calcium fluorescence of s-LNv dorsal fibers before, during, and after cooling at ZT6 (**A**) and ZT18 (**B**), respectively. $N = 6$ in (**A**), $N = 4$ for in (**B**). (**C**, **D**) The basal calcium fluorescence of s-LNv dorsal fibers before, during, and after heating at ZT6 (**C**) and ZT18 (**D**), respectively. $N = 4$ in (**C**), $N = 7$ in (**D**). (**E**, **F**) The maximum calcium fluorescence of s-LNv dorsal fibers to light pulse before, during, and after cooling at ZT6 (**E**) and ZT18 (**F**). $N = 6$ for ZT6, $N = 4$ for ZT18. $P < 0.0001$ for both 24 °C vs 15 °C and 15 °C vs 24 °C in (**E**). $P = 0.0036$ for 24 °C vs 15 °C, $P = 0.003$ for 15 °C vs 24 °C in (**F**). (**G**, **H**) The maximum calcium fluorescence of s-LNv dorsal fibers to light pulse before, during, and after heating at ZT6 (**G**) and ZT18 (**H**). $N = 4$ for ZT6, $N = 7$ for ZT18. $P = 0.0047$ for 22 °C vs 31 °C in (**G**). $P < 0.0001$ for both 22 °C vs 31 °C and 31 °C vs 22 °C in (**H**). Data information: Data are presented as means ± SEM. $^{**}P < 0.01$, $^{****}P < 0.0001$. One-way ANOVA with the Tukey correction to the P value was used.

 © The Author(s)

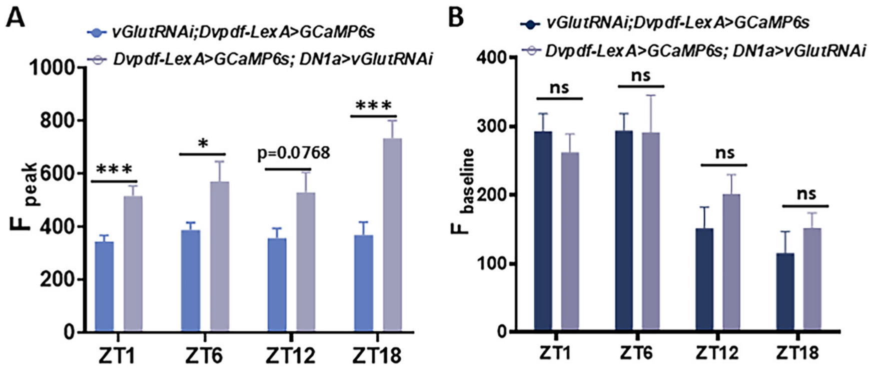

Figure EV5. DN1as exert glutamatergic inhibition to s-LNvs.

(**A**) The maximum calcium fluorescence (right) of s-LNv dorsal fibers to light pulse in wild-type control flies (blue) and DN1a-inhibited flies (purple). The number of wild-type control flies was $N = 8$ for ZT1 and ZT6, $N = 6$ for ZT12 and ZT18. The number of DN1a-inhibited flies was $N = 8$ for ZT1, ZT6, and ZT12, and $N = 6$ for ZT18. $P = 0.0005$ for ZT1, $P = 0.03$ for ZT6, $P = 0.0003$ for ZT18. (**B**) The basal calcium fluorescence of s-LNv dorsal fibers in wild-type control flies (blue) and DN1a-inhibited flies (purple). The number of wild-type control flies was $N = 8$ for ZT1 and ZT6, $N = 6$ for ZT12 and ZT18. The number of DN1a-inhibited flies was $N = 8$ for ZT1, ZT6, and ZT12, and $N = 6$ for ZT18. Data information: Data are presented as means ± SEM. $^*P < 0.05$, $^{**}P < 0.01$, $^{***}P < 0.001$. Two-tailed unpaired Student's *t* test was used.

© The Author(s)

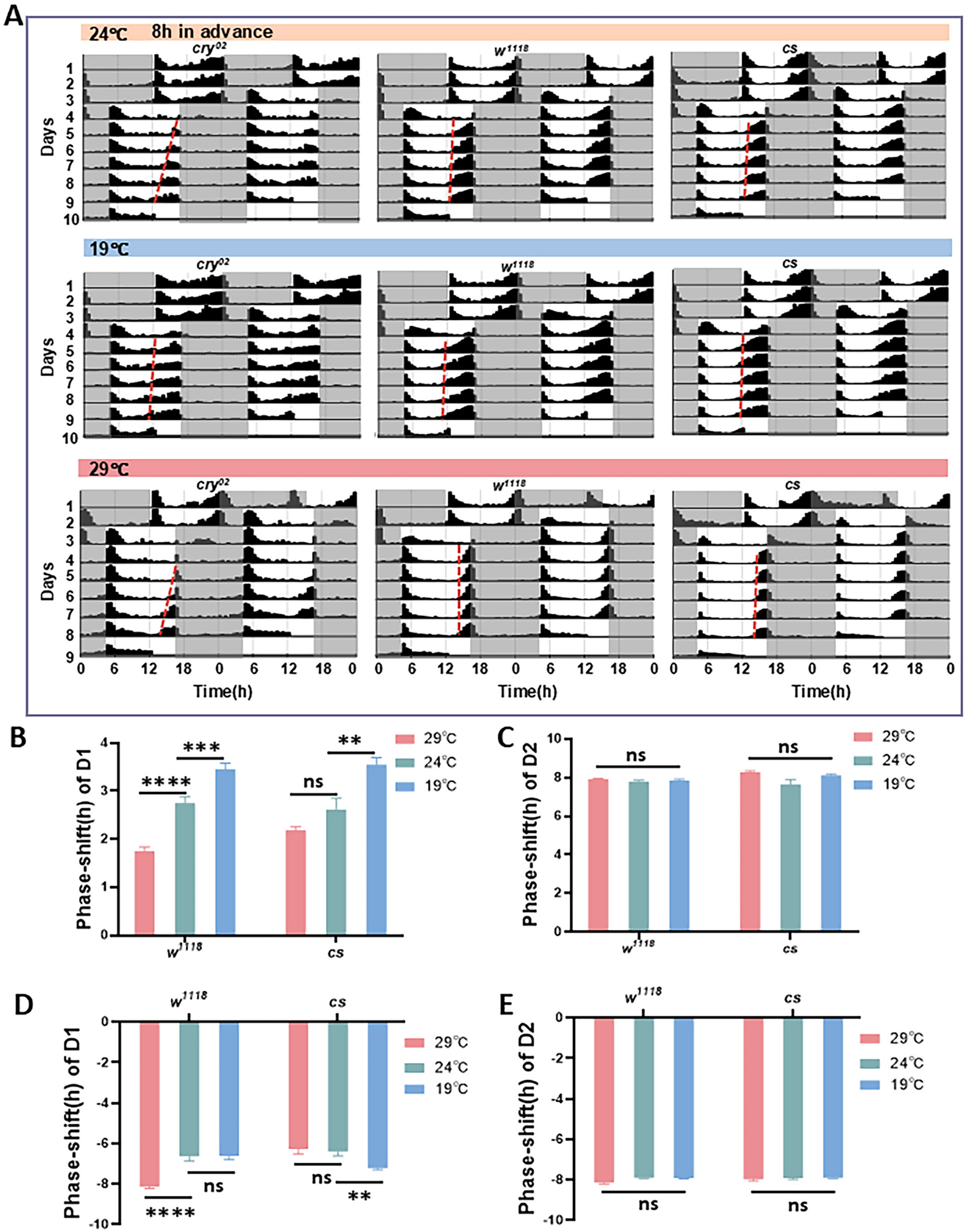
A
24℃ 8h in advance
cry02
w1118
cs
Days
19℃
29℃
Time(h)
B
Phase-shift(h) of D1
29℃
24℃
19℃
****
***
ns
**
w1118
cs
C
Phase-shift(h) of D2
ns
ns
D
Phase-shift(h) of D1
****
ns
ns
**
E
Phase-shift(h) of D2
ns
ns

 © The Author(s)

Figure EV6. Activity rhythms of other data were not included in Fig. 7.

(**A**) Under 12/2 h LD with an 8 h LD phase advance at 24 °C, 19 °C, and 29 °C. The gray area in the actograms indicates the dark phase. The red dashed line indicates the onset time of the evening activity peak. (**B**, **C**) Statistical quantification of the phase shift magnitude of wild-type flies on day 1 (**B**) and day 2 (**C**) after an 8 h LD phase delay at 24 °C, 29 °C, and 19 °C. The number of w^{1118} flies was $N = 24$ at 24 °C, $N = 40$ at 29 °C, N = 32 at 19 °C. The number of *cs* flies was $N = 22$ at 24 °C, $N = 34$ at 29 °C, $N = 31$ at 19 °C. For w^{1118} flies in (**B**), $P < 0.0001$ for 29 °C vs 24 °C, $P = 0.0002$ for 19 °C vs 24 °C. For *cs* flies in (**B**), $P = 0.0011$ for 19 °C vs 24 °C. (**D**, **E**) Statistical quantification of the phase shift magnitude of wild-type flies on day 2 (**D**) and day 2 (**E**) after an 8 h LD phase delay at 24 °C, 29 °C, and 19 °C. The number of w^{1118} flies was $N = 42$ at 24 °C, $N = 24$ at 29 °C, $N = 32$ at 19 °C. The number of *cs* flies was $N = 43$ at 24 °C, $N = 15$ at 29 °C, $N = 32$ at 19 °C. For w^{1118} flies in (**D**), $P < 0.0001$ for 29 °C vs 24 °C. For *cs* flies in (**D**), $P = 0.0085$ for 19 °C vs 24 °C. Data information: Data are presented as means ± SEM. **$P < 0.01$, ***$P < 0.001$, ****$P < 0.0001$. One-way ANOVA with the Tukey correction to the P value was used to assess the significance.

© The Author(s)
